# Development of guidance for statistical analysis plans (SAPs) for clinical trials

**DOI:** 10.1186/1745-6215-16-S2-O41

**Published:** 2015-11-16

**Authors:** Ashma Krishan, Deborah Stocken, Steff Lewis, Ed Juszczak, Caroline Dore, Paula Williamson, Doug Altman, Alan Montgomery, Mike Clarke, Carrol Gamble

**Affiliations:** 1 University of Liverpool, Liverpool, UK; 2 Newcastle University, Newcastle, UK; 3 University of Edinburgh, Edinburgh, UK; 4 University of Oxford, Oxford, UK; 5 MRC Clinical Trials Unit UCL, London, UK; 6 University of Nottingham, Nottingham, UK; 7 Queens University, Belfast, UK

## Background

ICH E9 guidelines recommend that a detailed SAP is prepared before breaking the trial blind. However, the guidelines do not provide specific guidance on SAP content.

## Methods

The project included: identification of existing SAP guidance; identifying and reviewing published SAPs; a survey of current practice within UKCRC registered Clinical Trial Units (rCTU) and a Delphi survey to establish consensus on SAP content. The Delphi survey included rCTU statisticians, CONSORT and SPIRIT guideline authors, experienced statisticians in the pharmaceutical industry, journal editors and regulators culminating in a face-to-face consensus meeting attended by experts from each demographic. These components informed the development of guidance for SAPs which subsequently underwent critical review by rCTU statisticians and experts from the consensus meeting, followed by piloting of the guidance document.

## Results

Apart from ICH E9, no existing SAP guidance was identified. A 100% response rate from the rCTU survey was achieved which highlighted diversity in current practice confirming support for developing comprehensive guidance. The Delphi survey reached consensus on 42% (46/110) of identified components. The expert panel agreed that 63 components should be included in the guidance. The guidance was written with support of examples identified from published and unpublished SAPs.

## Conclusions

A comprehensive SAP guidance document has been developed in collaboration with the network of rCTU statisticians with the aim of facilitating its acceptance and implementation. Although the guidance document is intended primarily for CTU adoption, given the representation involved in its developments it is anticipated that it will have wider applicability.

